# Virus-like and Virus Replicon Particles Targeting Multiple B-Cell Antigens Do Not Protect Against African Swine Fever Virus

**DOI:** 10.3390/vaccines14030285

**Published:** 2026-03-23

**Authors:** Kirill Lotonin, Obdulio García-Nicolás, Normann Kilb, Stefan Krämer, Xinyue Chang, Paul Engeroff, Kemal Mehinagic, Noelle Donzé, Francisco Brito, Matthias Liniger, Ilva Lieknina, Darja Cernova, Ieva Balta, Gabriela González-García, Paloma Rueda, Gert Zimmer, Charaf Benarafa, Nicolas Ruggli, Günter Roth, Kaspars Tars, Martin Bachmann, Artur Summerfield

**Affiliations:** 1 Institute of Virology and Immunology (IVI), Sensemattstrasse 293, 3147 Mittelhäusern, Switzerland; kirill.lotonin@students.unibe.ch (K.L.); obdulio.garcia-nicolas@ivi.admin.ch (O.G.-N.); kemal.mehinagic@ivi.admin.ch (K.M.); noelle.donze@ivi.admin.ch (N.D.); francisco.brito@unibe.ch (F.B.); matthias.liniger@ivi.admin.ch (M.L.); gert.zimmer@ivi.admin.ch (G.Z.); charaf.benarafa@ivi.admin.ch (C.B.); nicolas.ruggli@ivi.admin.ch (N.R.); 2 Department of Infectious Diseases and Pathobiology (DIP), University of Bern, 3012 Bern, Switzerland; 3 Graduate School for Cellular and Biomedical Sciences (GCB), University of Bern, 3012 Bern, Switzerland; 4 Multidisciplinary Center for Infectious Diseases, University of Bern, 3012 Bern, Switzerland; 5 BioCopy GmbH, 79312 Emmendingen, Germany; normann.kilb@biocopy.com (N.K.); guenter.roth@biocopy.com (G.R.); 6 Department of Rheumatology and Immunology, Inselspital, University of Bern, 3010 Bern, Switzerland; xinyuechang@ahau.edu.cn (X.C.); paul.engeroff@unibe.ch (P.E.); martin.bachmann@unibe.ch (M.B.); 7 Department for Biomedical Research (DBMR), University of Bern, 3012 Bern, Switzerland; 8 College of Veterinary Medicine, Anhui Agricultural University, Hefei 230036, China; 9 Latvian Biomedical Research & Study Centre, Ratsupites iela 1, LV 1067 Riga, Latvia; ilva@biomed.lu.lv (I.L.); darja.chernova@gmail.com (D.C.); ieva.balta@biomed.lu.lv (I.B.); kaspars@biomed.lu.lv (K.T.); 10 Gold Standard Diagnostics Madrid (GSD Madrid), 28037 Madrid, Spainpaloma.rueda@eu.goldstandarddiagnostics.com (P.R.)

**Keywords:** ASFV, protein microarray, subunit vaccine, VLP, viral vectored vaccine, VSV, B-cell antigens, challenge infection

## Abstract

**Background**: African swine fever virus (ASFV) causes a fatal hemorrhagic disease in domestic pigs and wild boars. While live attenuated vaccines (LAVs) provide protection, their use raises safety concerns. Therefore, the aim of the present study was to identify viral B-cell antigens associated with protection and to test their potential using highly immunogenic vaccine delivery platforms. **Methods**: We employed a microarray of 169 ASFV proteins expressed in a cell-free prokaryotic system to identify immunodominant antigens using sera from immune pigs. Six structural proteins were selected and formulated into AP205 virus-like particles (VLPs). Additionally, replication-defective vesicular stomatitis virus (VSV)-based vaccine candidates expressing glycosylated CD2v and EP153R proteins were generated. Three groups of specific pathogen-free pigs were immunized with either VLP- or VSV-based vaccines and challenged with the virulent ASFV Georgia 2007 strain. Control groups included pigs immunized with the attenuated ASFV Estonia 2014 strain and a naïve group. **Results**: Most vaccine candidates induced detectable antibody responses against target ASFV proteins. However, neither VLP- nor VSV-based vaccines provided protection, as clinical scores, hematology, cytokine responses, and viremia levels were similar to those in the negative control group. In contrast, only the ASFV Estonia 2014 strain elicited a robust T-cell response and protective immunity. **Conclusions**: These findings highlight the challenges in identifying protective B-cell antigens of ASFV and emphasize the pivotal role of cellular immunity in mediating protection.

## 1. Introduction

African swine fever virus (ASFV) causes a deadly hemorrhagic disease affecting both domestic pigs and wild boars. ASFV is a major concern for global swine production, with outbreaks leading to massive economic losses and significant threats to food security [1]. The virus spreads through direct contact to infected animals, contaminated feed, equipment, vehicles and contaminated pork products. ASFV control is particularly challenging due to the virus’s ability to persist in the environment, its circulation in wild boars, lack of biosecurity in the field, as well as the lack of vaccines [2,3,4,5].

Although efficacious live attenuated vaccines (LAVs) against ASFV have been developed, their broader application is constrained by safety concerns [6,7]. The potential for reversion to virulence, the danger of recombination with circulating field strains, as well as the residual pathogenicity in pregnant or immunosuppressed animals present significant challenges [8]. As a result, the development of safer vaccines is highly desired. Despite significant research efforts, alternative vaccine types, including subunit, DNA, and virus-vectored vaccines, have not yet demonstrated sufficient protection, especially against the highly virulent genotype II strains responsible for the ongoing ASF pandemic [9,10].

The complexity of the ASFV genome, its large size, and the variability of antigens expressed during infection complicate the identification of protective immune responses. In addition, ASFV encodes a wide array of proteins that interact with the host immune system in complex ways, making it difficult to pinpoint the precise antigens that mediate protective immunity [2,10,11]. Previous studies have demonstrated that both cellular and humoral immune responses contribute to protection [12,13]. However, B-cell-mediated immunity, the role of antibodies in neutralizing the virus and protective antigens remain controversial and underexplored [14,15,16,17,18].

Accordingly, this study was initiated to identify potential new antigenic targets for protective antibody responses, as well as for testing delivery systems known for their high immunogenicity toward induced antibody responses. As a first platform, we employed virus-like particles (VLPs) representing non-infectious, nanoparticle-based vaccines that are highly immunogenic for antibody responses due to their mimicry of viral particulate structures and dense repetitive epitope presentation [19]. The VLPs were based on the AP205 bacteriophage protein engineered to display viral antigens expressed in *E. coli* [20]. For highly glycosylated antigens, we used a non-spreading virus replicon particle (VRP) based on G-protein-deleted vesicular stomatitis virus (VSV) as a second platform, which was previously described to induce potent antibody responses in pigs [21].

Antigens were selected based on the following selection criteria. The first selection targeted novel B-cell antigens, which we selected based on their recognition only by serum from pigs that were protected (products of B169L and H171R). Our hypothesis was that this would indicate their involvement in protection. To identify such antigens, we employed a chip-based protein microarray to immobilize 169 ASFV proteins printed from a genomic DNA template [22]. The proteins were then probed using immune sera from pigs that had developed varying levels of protective immunity against ASFV infection in a previous study [23]. The second selection criterion was based on the described localization of antigens with a focus on antigens located in the outer membrane of the virions (EP402R (CD2v)) or on infected cells (O61R (p12), KP177R (p22), EP153R) [10,24,25]. CD2v and EP153R were also selected based on their involvement in hemadsorption, and their relatively high genetic heterogeneity amongst ASFV genotypes, which indicates that they could be targeted by functionally relevant antibodies [26,27,28]. This selection was based on the hypothesis that antibodies targeting such proteins could mediate functions such as inhibition of virus attachment and entry, antibody-dependent cellular cytotoxicity (ADCC) or phagocytosis (ADCP) [17].

The VLP- and VSV-based vaccine candidates were evaluated in a vaccine trial in pigs that were challenged with the highly virulent genotype II ASFV Georgia 2007 (ASFV-G) strain. Although the animals developed antibody responses against most of the viral antigens used, they were not protected against the challenge infection.

## 2. Materials and Methods

### 2.1. DNA Isolation and Protein Chip Production

The microarray-based antigen identification is schematically represented in Supplementary Figure S1A. DNA of ASFV Georgia 2007/1 (GenBank accession no. FR682468.2) was extracted from supernatants of infected macrophages using the NucleoSpin RNA Virus kit (Macherey-Nagel, Düren, Germany). Primer pairs for 199 ORFs (annotated or hypothetical from NCBI database) of ASFV were designed. After amplification of the ORFs, the PCR products were purified using AMPure XP magnetic beads (Beckman Coulter, Brea, CA, USA). Quality control of the PCR products was performed using agarose gel electrophoresis. In case smear or double bands were observed, the products were purified by gel purification with the QIAquick gel extraction kit (Qiagen, Hilden, Germany). All PCR products were then assembled with two additional modules (promoter + start codon + HA-tag upstream; modified SNAP-tag + stop codon downstream) using overlap extension PCR. During this process, the linear templates were also modified with a C6 amino linker at the downstream end (5’ on the complementary strand). Next, the templates were printed on a 5k hex PDMS (polydimethylsiloxane) chip with a multilayer surface chemistry. The top layer was PDITC (p-Phenylene di-isothiocyanate), which covalently binds the C6 amino linker. The printing was performed by INTER-ARRAY (Bad Langensalza, Germany), with samples in a 150 mM Na_2_HPO_4_ buffer (pH 8). A detailed protocol for PDMS chip production and a description of the PDITC chemistry were previously reported [22].

The protein copy process was based on a published protocol [29]. The chips were rehydrated in Petri dishes containing a towel moistened with 150 mM Na_2_HPO_4_ buffer, sealed with parafilm and incubated at 30 °C for 10 min. Afterwards, the chips were baked at 60 °C for 15 min, washed with PDMS blocking solution (PBS, 5% ethanolamine, 10 mg/mL BSA, pH 8), and then placed into a slide holder filled with the same blocking solution. Next, the entire slide holder was placed in a desiccator, which was frequently tapped on the bench to release air bubbles. After 30 min, the chips were rinsed with deionized (DI water, dried using a nitrogen (N_2_) stream, and stored under vacuum at 4 °C. For expression, 0.5 µL of the expression mix was added to each chip, and the capture slide was carefully placed on top. The remaining vacuum ensured that the cavities were filled with the expression mix. The assembled chip/slide sandwiches were then secured using a clamp to maintain contact. The slides were incubated at 37 °C for 90 min or at 25 °C for 2 days (ALiCE system). Expression was verified by measuring the fluorescence of the GFP controls (Supplementary Figure S1B). In total, 169 ASFV proteins were expressed using *E. coli* lysate in a cell-free manner. The expression was confirmed by detecting a signal from the HA-tag (Supplementary Figure S1C,D). After expression, the chip/slide sandwiches were placed on a cooled aluminum block and carefully opened. The glass slide (protein capture slide) was quickly rinsed with DI water, dried with an N_2_ stream, and placed into a slide holder. The slide holder was vacuum-sealed and stored at 4 °C until single-color reflectometry (SCORE) followed by conversion to arbitrary units (A.U.), as previously described [22,30].

### 2.2. Antigen Screening

The assay involved five serum samples from farm pigs and five from SPF pigs, all collected on day 151 after immunization with the attenuated ASFV Estonia 2014 (ASFV-Est14) strain. In addition, two samples were obtained from SPF pigs immunized with the ASFV-Est14 strain and subsequently challenged with the virulent ASFV Armenia 2008 strain, collected on day 21 after challenge. The immunization-challenge experiment was previously described [23]. As negative controls, we used sera from three non-infected SPF pigs. In total, 89 measurements on the microarrays were performed, of which 73 were employed for data evaluation. The scanner intensity of 33% was used to carry out measurements. The difference in antigenic signal intensity (A.U.) to the negative control was determined to create volcano plots. The *p*-value cutoff was set to 0.05.

### 2.3. Production of Proteins and AP205 VLP

All DNA constructs for protein expression in *E. coli* were ordered via gene synthesis in the pETDuet-1 vector and inserted between NcoI and PstI restriction sites using *E. coli* optimized codons. The exact amino acid sequences of ASFV proteins were taken from the African swine fever virus isolate China/2018/AnhuiXCGQ full genome. *E. coli* BL21 (DE3) cells were transformed with plasmids containing the corresponding protein sequences, and individual colonies were grown overnight at 37 °C in LB media without shaking. The obtained cultures were used to grow *E. coli* biomass in 2xTY media in flasks until an OD600 of 0.6–0.8 was reached. IPTG was added to a 1 mM final concentration, and flasks were further incubated at 37 °C for 4 h. Cells were collected by centrifugation and lysed by sonication. Standalone proteins were purified by immobilized metal affinity chromatography (IMAC) using HisTrap columns, followed by ion exchange using Q Sepharose. The AP205 VLP constructs were purified by gel filtration with Sepharose 4FF, followed by ion exchange using Fractogel DEAE (M). All sequence information for the antigen employed for the VLP-based vaccines is given in Supplementary Figure S2. VLP-fusion proteins were analyzed by SDS-PAGE (Supplementary Figures S3 and S4). Production of VLP was previously described [31].

### 2.4. Production of the CD2v Protein and Chemical Coupling to AP205 VLP

CD2v was produced as a genetic fusion to the extracellular domain of CD2v with the Fc domain of human IgG1 (PDB: 4CDH_A) in Expi293 cells using ExpiFectamine 293 transfection kit (A14524, Thermo Fisher Scientific, Waltham, MA, USA). Cell supernatants containing CD2v-Fc protein were collected and purified with a protein G column (GE29-0485-81, Cytiva, Marlborough, MA, USA). The purified CD2v-Fc was buffer-exchanged into PBS by dialysis. Next, CD2v-Fc was chemically coupled to AP205d by succinimidyl 6-(β-maleimidopropionamido) hexanoate (SMPH). To this end, AP205d was mixed with SMPH at a molar ratio of 1:5 and incubated at room temperature for 30 min. Excess SMPH was removed by passing the mixture through a 7 K MWCO Zeba Spin desalting column (A57759, Thermo Fisher Scientific, USA). Meanwhile, CD2v-Fc was incubated with the mild reducing reagent TCEP at a molar ratio of 1:10. Next, the reduced CD2v-Fc was mixed with AP205d VLPs at a molar ratio of 1:1 and incubated at room temperature for 3 h with shaking at 400 rpm. The coupled product VLP-CD2v-Fc was then loaded into an SDS-PAGE gel to check the coupling efficiency.

### 2.5. Chemical Coupling of the p22 Protein to AP205 VLP

AP205 VLPs (1 mg/mL, in PBS) were incubated with a tetrazine-NHS linker (1 mM final concentration) for 1 h at room temperature, and excess linker was removed by a desalting column. The p22 protein (1 mg/mL in PBS) was incubated with a BCN-NHS linker (1 mM final concentration) for 1 h at room temperature, and excess linker was removed by a desalting column. Linker-labelled VLPs and p22 were mixed in a volume ratio of 1:1 and left at room temperature for 1 h. Excess p22 was removed by dialysis (overnight) using 300 kDa molecular weight cutoff dialysis tubes. The coupled product VLP-p22 was then loaded into an SDS-PAGE gel to check the coupling efficiency.

### 2.6. Electron Microscopy

Purified VLP samples (1.0 mg/mL) were adsorbed onto carbon-formvar-coated copper grids, negatively stained with a 1% aqueous solution of uranyl acetate and visualized using a JEM-1230 electron microscope (JEOL Ltd., Akishima, Japan) operated at 100 kV.

### 2.7. Mouse Immunization

BALB/c mice (female, 8-week-old) were used to assess the immunogenicity of the generated VLPs. Each group was assigned 5 mice. Thirty micrograms of VLPs were administered via subcutaneous injection on days 0 and 28. For VLP-CD2v, the dose was 40 μg per injection. Blood samples were collected from the tail vein on days 14, 21, 28, 35, and 42, from which serum was obtained by centrifugation. Mice were euthanized by CO_2_ atmosphere either on day 42 (VLP-CD2v group) or on day 49 (other groups). Serum samples were kept at −20 °C for further assays.

### 2.8. ELISA-Based Assessment of Antibody Responses to ASFV Antigens on VLPs in Mice

For ELISA plate coating, standalone variants of B117L, B169L and H171R proteins were produced in *E. coli* by expressing the corresponding genes engineered to include an N-terminal 6xHis-tag. The B117L protein was produced as a soluble fusion protein to MBP. In the case of p22, the protein was already produced in a standalone form for coupling to VLPs; thus, the same preparation was used for ELISAs. In the case of p12, a synthetic peptide corresponding to amino acid residues 1–12 was used for ELISAs. For CD2v, a fusion protein with a SUMO-tag was expressed in mammalian cells. ELISAs were performed to determine the antibody levels in the sera of immunized mice. Briefly, half-area 96-well plates were coated with 1 mg/mL of recombinant proteins at 4 °C overnight. Then, PBS with 0.15% casein was added and incubated at room temperature for 2 h, followed by washing with PBS for 3 times. Next, serum samples were 4-fold serially diluted from 1:20 with PBS-0.15% casein and incubated at room temperature for 1 h. After washing with PBST 3 times, plates were incubated with goat anti-mouse IgG-HRP (Jackson ImmunoResearch, Ely, UK) for 1 h at room temperature. Finally, TMB was added and incubated for 5 min, after which an equal volume of stop solution (1 M H_2_SO_4_) was added. The plates were read under OD_450_. Antibody titers were calculated as a dilution fold reaching half of OD_max_.

### 2.9. Generation of Recombinant VSV-Vectored Vaccine Candidates

The EP402R (CD2v) and EP153R genes were amplified by PCR from the ASFV Georgia 2007/1 genomic DNA using Phusion high-fidelity DNA polymerase (Life Technologies, Zug, Switzerland, cat. no. F530S). The gel-purified PCR products were treated with MluI and NheI endonuclease restriction enzymes and ligated into the corresponding treated pVSV*ΔG(HA) plasmid [32]. The resulting plasmids pVSVΔG(EP153R) and pVSVΔG(EP402R) encode the VSV genome (serotype Indiana, GenBank accession no. J02428.1) with the VSV glycoprotein (G) gene replaced by the EP153R or the EP402R gene (Supplementary Figure S2; ASFV G 2007/1, GenBank: FR682468.2). The GFP gene in the pVSV*ΔG(HA) plasmid was also removed when the ASFV genes were inserted into the vector.

The G protein-deficient VSV vectors were generated as previously described [32]. Briefly, BHK-G43 cells [33] were infected for 1 h with Modified Vaccinia Ankara virus expressing the T7 phage RNA polymerase (MVA-T7) using a multiplicity of infection of 3 focus-forming units (ffu)/cell. Thereafter, the cell culture medium was replaced by GMEM (Thermo Fisher Scientific) containing 5% FBS and 10^−9^ M mifepristone (Merck, Darmstadt, Germany). The cells were subsequently transfected with either the pVSVΔG(EP153R) or the pVSVΔG(EP402R) plasmid along with three plasmids encoding the VSV N, P, and L proteins. At 24 h after transfection, the BHK-G43 cells were trypsinized and seeded together with an equal number of non-transfected BHK-G43 cells. The cells were incubated at 37 °C for 24 h in the presence of 10^−9^ M mifepristone. The supernatant was collected and clarified by centrifugation (3000 rpm, 10 min, 4 °C) and subsequently passed through a 0.2 µm pore size filter to deplete MVA-T7. Infectious VSVΔG-CD2v and VSVΔG-EP153R were propagated twice on mifepristone-treated BHK-G43 cells and stored in aliquots at −70 °C in the presence of 10% FBS. Infectious virus titers were determined on BHK-21 cells by staining with a monoclonal antibody (Mab 23H12) directed against the VSV matrix (M) protein (Kerafast, Boston, MA, USA, cat. no. EB0011), and anti-mouse IgG conjugated with AlexaFluor-488 (Thermo Fisher Scientific, cat. no. A28175). Infected cells were detected by fluorescence microscopy. Virus titers were expressed as ffu/mL.

### 2.10. Detection of Vector-Driven ASFV Protein Expression

To confirm the expression of ASFV proteins CD2v and EP153R in mammalian cells, three cell lines were used. HEK-293 (ATCC CRL-1573™), Vero (ATCC CCL-81™), and IPKM cells (kindly received from the Division of Transboundary Animal Disease Research at the National Institute of Animal Health, Kodaira, Tokyo, Japan; [34]) were infected with VSVΔG-CD2v and VSVΔG-EP153R at MOI = 2. VSV expressing GFP was used as a negative control. After 20 h of incubation at 37 °C, the cells were trypsinized and washed. Cytofix/Cytoperm kit (BD Biosciences, San Diego, CA, USA) was used for fixation and permeabilization according to the manufacturer’s instructions. Next, the cells were stained with ASFV-positive serum diluted 1:500 (collected on day 23 post-challenge from an SPF pig experimentally immunized with ASFV-Est14 and later challenged with the Armenia 2008 strain) followed by anti-swine IgG coupled with biotin (A100-104B, Bethyl Laboratories). Finally, SAV-BV421 (563259, BD Biosciences, USA) was added, and samples were acquired on FACSCanto II flow cytometer (BD Biosciences, USA). The data was analyzed using FlowJo v10 software (BD Biosciences) following the gating strategy shown in Supplementary Figure S5A.

### 2.11. Vaccination-Challenge Experiment in Pigs

A total of 25 male and female Large White domestic pigs (12 weeks of age; ~30 kg) were obtained from the IVI SPF facility. The animals were moved to the BSL3-Ag containment facility of the IVI and randomly assigned to five groups (5 pigs/group). VLP-based vaccines were formulated with Montanide 28R (Seppic, La Garenne Colombes, France), and each animal was given 2 mL per intramuscular injection. After 7 days of acclimatization, pigs from the first group received 40 μg of VLP-CD2v. The animals from the VLP mix group received a mixture of five VLP-based constructs (VLP-p12, VLP-p22, VLP-B117L, VLP-B169L, and VLP-H171R; 100 μg of each one per animal). The pigs in the negative control group received 100 μg of empty AP205 VLP. VSV vectors were diluted in GMEM BHK-21 medium (Gibco, USA). Each animal from the VSV vaccine group received a mixture of VSVΔG-CD2v and VSVΔG-EP153R in a 3 mL final volume (10^8^ ffu of each vector). The animals in the positive control group were immunized oronasally with the attenuated ASFV-Est14 using a dose of 10^6^ TCID_50_ per animal in a final volume of 5 mL (in MEM medium, Gibco, USA). The animals from VLP and VSV groups received two vaccine boosts on days 21 and 42 post-immunization (pi), respectively (100 μg of each VLP construct or 10^8^ ffu of each VSV vector per pig). One animal in the positive group did not survive the infection and had to be euthanized on day 24. The remaining 24 pigs were challenged oronasally with the highly virulent ASFV-G strain (10^6^ TCID_50_/animal) on day 65 pi, which corresponds to day 0 post-challenge (pc). Body temperature and clinical parameters were assessed daily by a veterinarian based on a clinical score checklist previously described [35]. Pigs were euthanized as soon as the pre-defined discontinuation criteria were reached. This was defined as a cumulative score of 18 or a score of 3 in one of the following parameters: liveliness, body tension, breathing, walking or skin. All animals in the VLP and VSV groups were sacrificed by day 7 pc after reaching a cumulative score of 18. Survived animals from the ASFV-Est14 group were kept in a stable state until day 191 pi (126 pc). Rectal temperature and clinical scores were recorded daily by a veterinarian. Blood samples were taken for viremia monitoring, hematological analysis, PBMC isolation, as well as measurements of cytokines and antigen-specific antibodies. Additionally, spleen, liver and gastrohepatic lymph nodes were collected for virus DNA quantification.

### 2.12. Virus Stocks and Viremia Quantification

ASFV-Est14 was kindly provided by Sandra Blome and Martin Beer (Friedrich-Loeffler-Institut, Greifswald—Insel Riems, Germany). ASFV-G belonging to the same genotype is an in-house strain of the Institute of Virology and Immunology. It was isolated from an SPF pig that had been orally infected with spleen homogenate from the 2007 ASFV outbreak in Georgia. Virus titers for inoculation were determined by calculating the 50% TCID_50_ using AEC staining for p72 capsid protein in IPKM cells [34]. For viral load quantification, ASFV DNA was isolated from samples using the NucleoMag VET kit (Macherey-Nagel, Düren, Germany) and the Kingfisher Flex extraction robot (Thermo Fisher Scientific, USA) according to the manufacturer’s instructions. DNA extractions were performed with 200 μL of EDTA blood, serum or organ homogenates in RA1 lysis buffer. Further, qPCR was performed according to the published protocol [36], followed by quantification of genome equivalents [23]. Standards were analyzed in triplicate, and samples in duplicate.

### 2.13. Hematology and Cytokine Analyses

Differential blood cell counts from EDTA blood samples were measured using the Vetscan HM5 hematology analyzer (Zoetis, Leatherhead, UK). The following serum cytokines were measured by commercial ELISA: IL-1α, IL-1β (RayBiotech, Peachtree Corners, GA, USA), IL-1RA, IL-8 (R&D Systems, Minneapolis, MN, USA), and IFN-α (in-house assay using the monoclonal antibodies K9 and F17 that were kindly provided by Dr. B. Charley, INRAE, Jouy-en-Josas, France [37]).

### 2.14. ELISA-Based Assessment of Antibody Responses to ASFV Antigens in Pigs

To detect antigen-specific antibodies after vaccination in pigs, we used the same recombinant proteins as in the experiments with mice sera. In brief, 96-well ELISA plates were coated with 2 mg/mL of the proteins at 4 °C overnight. The plates were then blocked for 2 h with PBS-1% BSA and washed with PBS-Tween 0.05% 5 times. After that, serum samples were 10-fold serially diluted from 1:20 with PBS-0.1% BSA and incubated at room temperature for 2 h. After washing with PBS-Tween 0.05% 5 times, plates were incubated with goat anti-swine IgG-HRP (Jackson ImmunoResearch, UK) for 1 h at room temperature. Finally, substrate buffer containing OPD tablets (34006, Thermo Scientific, USA) and 30% H_2_O_2_ was added to the plates. After 15 min incubation, OD measurements were taken at 450 nm. The antibody response against the EP153R protein was detected using an indirect ELISA, with plates coated with the extracellular domain of the genotype II protein (Gold Standard Diagnostics Madrid, Madrid, Spain). The antigen production and ELISA protocol used were developed during the European Union–funded VACDIVA project (No. 862874) and were described in detail previously [38].

### 2.15. IFN-γ ELISpot

Freshly collected peripheral blood mononuclear cells (PBMC) were isolated using Ficoll-Paque 1.077 g/L density centrifugation and used to quantify cytokine production in response to the live virus. Briefly, 96-well MultiScreen PVDF plates (Merck) were coated with a capture antibody (559961, BD Biosciences) at 0.83 μg/mL in sterile PBS, incubated overnight at 4 °C and blocked for 1 h at 37 °C with 0.5% BSA diluted in PBS. PBMC were counted and plated in ELISpot plates at 5 × 10^5^ cells/well (triplicate per condition) in AIM-V medium (Gibco, USA). Cells were restimulated either with ASFV-Est14 (MOI = 0.1) or with supernatant of mock-treated IPKM cells. After 36 h of incubation at 37 °C with 5% CO_2_, the plates were stained with a biotinylated detection antibody (559958, BD Pharmingen, USA) followed by streptavidin-HRP (Agilent, Santa Clara, CA, USA) and TMB substrate (Mabtech, Stockholm, Sweden). The plates were scanned using ImmunoSpot S5 UV analyzer (Cellular Technology Ltd., Shaker Heights, OH, USA).

### 2.16. Intracellular Cytokine Staining

To analyze production of IFN-γ and TNF by several immune cell subsets, freshly isolated PBMC were seeded in U-shaped 96-well plates at 5 × 10^5^ cells/well in AIM-V medium (Gibco, USA) and restimulated with live ASFV-Est14 (MOI = 0.1/cell) or mock treated for 18 h at 37 °C with 5% CO_2_. Brefeldin A (Thermo Fisher Scientific) was added 4 h before cell harvesting at a 1:1000 dilution. The antibody staining protocol was performed as previously described [39]. Antibodies and secondary reagents are listed in Supplementary Table S1. Free binding sites of secondary antibodies were blocked with mouse IgG (Jackson ImmunoResearch, UK). Live/Dead Aqua stain kit (Thermo Fisher Scientific) was used to discriminate and exclude dead cells from the analysis. Cytofix/Cytoperm kit (BD Biosciences) was used for fixation and permeabilization according to manufacturer’s instruction. The samples were acquired on FACSCanto II flow cytometer (BD Biosciences, USA). The data was analyzed using FlowJo v10 software (BD Biosciences) following the gating strategy shown in Supplementary Figure S6.

### 2.17. Statistical Analysis

GraphPad Prism version 10 (GraphPad Software, San Diego, CA, USA) was used for data analysis and the generation of figures. The statistical tests used to evaluate the data are indicated in the respective figure legends. Outliers were tested using the ROUT method (Q = 1%). Images showing experiment setups were designed in BioRender (BioRender, Toronto, ON, Canada).

## 3. Results

### 3.1. Identification of B-Cell Antigens Using Protein Microarray

**Figure 1:**
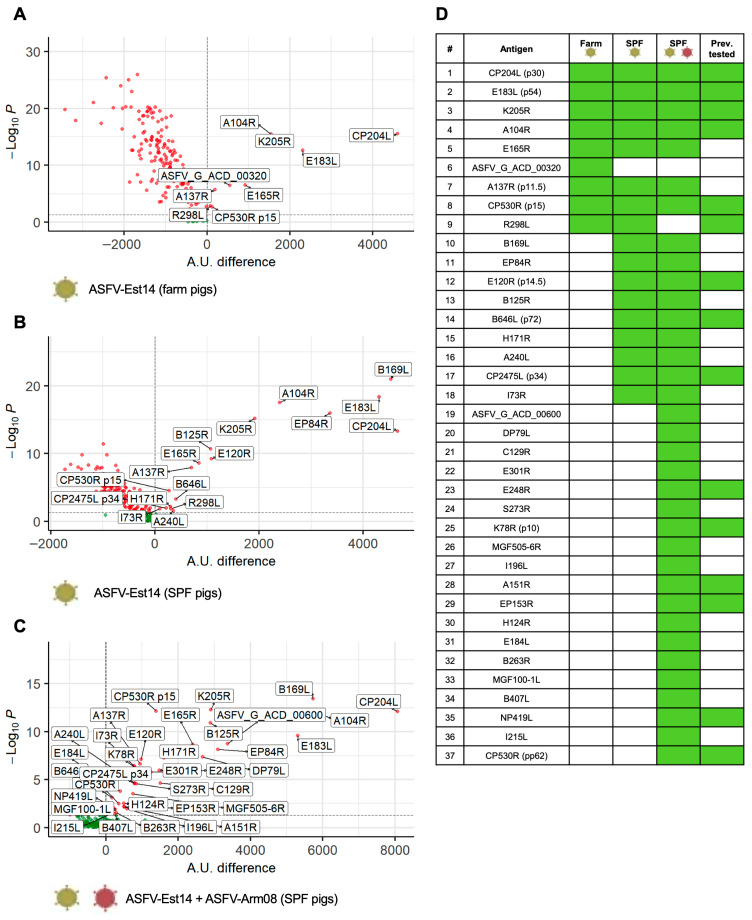
Screening of viral antigens using protein microarray. (**A**–**C**) Volcano plots showing microarray results for the detection of ASFV proteins using immune sera from farm or SPF pigs immunized with the attenuated ASFV-Est14 strain (**A**,**B**), respectively, or from ASFV-immunized SPF pigs challenged with the virulent ASFV Armenia 2008 strain (ASFV-Arm08) (**C**). The *y*-axis represents −log10(p), while the *x*-axis represents the scan signal for antibody-antigen binding in arbitrary units (A.U. difference). (**D**) List of identified ASFV antigens, specifying whether they were previously tested as subunit vaccine components.

The pipeline for the identification of antigenic ASFV proteins involved several steps, as depicted in Supplementary Figure S1A. First, an expressible DNA microarray PDMS chip was produced. Next, a protein chip copy was generated using cell-free *E. coli* lysate-based expression. In total, 169 HA-tagged ASFV proteins were successfully expressed on microarrays (Supplementary Figure S1C). Serum samples from naïve or immune pigs, either of farm or SPF origin, stored from the previous infection experiment [23], were then used to screen binding of antibodies to viral proteins using a microfluidic system (Supplementary Figure S1D). The obtained values were used to generate volcano plots illustrating identified antigens (Figure 1A–C).

As shown in Figure 1A–D, the number of proteins recognized by the immune sera of the SPF pigs was higher compared with farm pigs following infection with the attenuated ASFV-Est14. The nine hits identified in the farm group included highly immunogenic antigens previously tested in subunit vaccine formulations [9,10], such as CP204L (p30), E183L (p54), K205R, and A104R, as well as less studied proteins E165R and A137R (Figure 1A). By using sera from SPF pigs, additional antigens were detected, including several that have not previously been reported as vaccine components: B169L, EP84R, B125R, H171R, A240L, and I73R (Figure 1B). Following challenge infection with the ASFV Armenia 2008 strain (Arm08), the number of proteins recognized by the sera further increased (Figure 1C). Although they include established antigens such as EP153R [27] and CP530R (pp62) [40], most have not yet been assessed in vaccine formulations.

### 3.2. VLP Generation and Immunization of Mice

We selected six ASFV antigens for the generation of nanoparticle vaccine candidates based on the criteria explained in the Introduction. O61R (p12) was selected based on its possible role in virus attachment to cells, and its reported presence in the virion’s outer envelope [25,41], although this has not been confirmed [10,24,25]. CD2v was selected as an established protein of the outer envelope and its expression on the surface of virus-infected cells [24]. KP177R (p22) was also selected based on its expression on the surface of infected cells, although it is an inner membrane protein [24,42]. Besides that, we picked three exploratory proteins, which, to our knowledge, had not been tested in vaccine formulations. B169L and H171R were selected as proteins recognized by SPF serum only. B169L and B117L represent structural proteins localized in the inner viral membrane and have been reported to have viroporin functions possibly involved in viral entry to the cytoplasm [2], a process that may be targeted by antibodies. The amino acid sequences of the selected ASFV antigens are shown in Supplementary Figure S2.

VLPs were decorated with the selected antigens by two different methods—genetic fusion and chemical coupling. In the case of genetic fusion, we used a modified version of AP205 VLP, namely AP205d, in which two AP205 coat protein sequences were joined in tandem to create a covalent coat protein dimer. AP205d has been previously shown to better accommodate the insertion of foreign amino acid sequences compared with regular AP205 VLP [43]. Full-length B117L, B169L, H171R proteins and residues 1–12 from p12 were exposed on AP205d VLP by fusing respective protein genes to the C-terminus of the second repeat of the AP205 coat protein gene. In all cases, we obtained soluble VLPs with displayed antigens. However, the genetic fusion approach was unsuccessful with p22 and CD2v proteins, as it resulted in insoluble products. Instead, we used chemical coupling to expose p22 and CD2v on AP205 VLPs. The CD2v protein was only produced in inclusion bodies in *E. coli*, and attempts at re-folding were unsuccessful. As a result, we transitioned to a mammalian expression system. CD2v was produced as a genetic fusion to the Fc domain of human IgG1 and subsequently coupled to AP205 VLP by SMPH. For the p22 protein, which was produced in a soluble form in *E. coli*, a different approach was employed, involving two separate linkers. First, p22 was treated with a lysine-specific linker, with a succinimide group at one end and a cyclooctyne moiety at the other. AP205 VLPs were treated with a lysine-specific linker, featuring a succinimide group at one end and a tetrazine group at the other. Subsequently, both proteins were linked using click chemistry, joining the cyclooctyne and tetrazine moieties, a method previously employed in a similar context [44].

**Figure 2:**
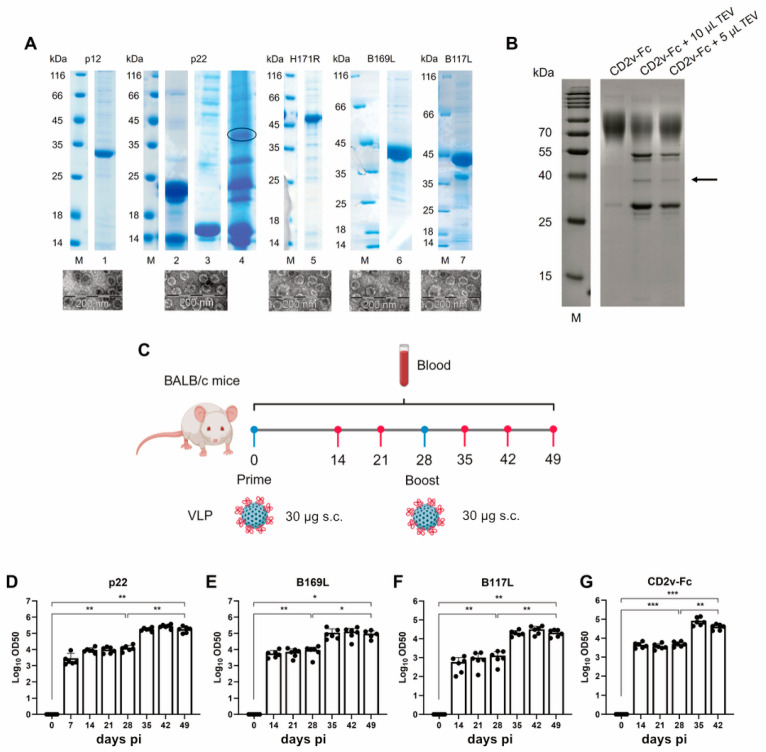
Characterization of VLP vaccines. (**A**) SDS-PAGE and electron microscopy analysis of purified AP205d-fusion proteins: p12 (lane 1), H171R (lane 5), B169L (lane 6), and B117L (lane 7). The p22 protein was produced separately (lane 2) and chemically coupled to AP205 VLP (lane 3). The coupled product is encircled in lane 4. M indicates the molecular weight marker. The electron micrographs of the corresponding VLP are shown below. (**B**) SDS-PAGE analysis of CD2v-Fc VLP after TEV protease cleavage of the Fc region, allowing visualization of the CD2v portion (indicated by an arrow). (**C**) Overview of VLP immunization in mice. (**D**–**G**) Antigen-specific IgG antibody titers in serum samples obtained at the indicated days pi from mice immunized with VLP. Data represent the mean ± standard deviation of measurements (6 mice/group). Statistical analysis was performed using paired *t* test; * *p* < 0.05, ** *p* < 0.01, *** *p* < 0.001.

SDS-PAGE gel and electron microscopy (EM) images of the five VLPs used for vaccination are shown in Figure 2A, Supplementary Figures S3 and S4. In addition, the CD2v-Fc-fusion protein was treated with TEV protease and loaded onto the gel to confirm the right size of the CD2v standalone protein (Figure 2B). After the production of the vaccine candidate, BALB/c mice were immunized with VLP containing p22, B117L, B169L, or CD2v antigen (Figure 2C). Having received two doses of the vaccine with a four-week interval, mice developed prominent antigen-specific antibody titers, particularly after the second VLP dose (Figure 2D–G).

### 3.3. Production of VSV-Vectored Vaccine Candidates

**Figure 3:**
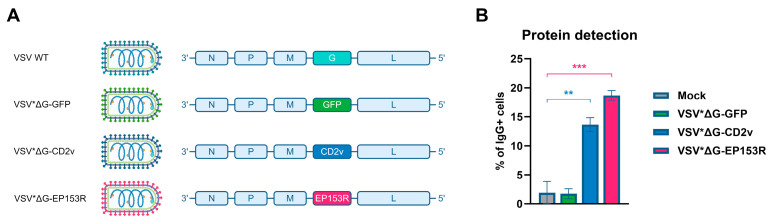
Generation of recombinant VSV vectors. (**A**) Genome maps of wild type (WT) and recombinant VSVs. The genome of VSV WT encodes 5 proteins: nucleoprotein (N), phosphoprotein (P), matrix protein (M), glycoprotein (G), and large polymerase protein (L). In the vaccine vectors VSVΔG-CD2v and VSVΔG-EP153R, the G glycoprotein was replaced with CD2v and EP153R, respectively. In the control vector, it was replaced with GFP. (**B**) Detection of ASFV protein expression in VSV-infected Vero cells using ASFV-positive serum. Data represent the mean ± standard deviation of measurements with duplicates. Statistical comparison was performed by one-way ANOVA with Dunnett’s correction for multiple comparisons; ** *p* < 0.01, *** *p* < 0.001.

The two serotype-specific proteins of ASFV, CD2v (EP402R) and C-type lectin (EP153R), which have been proposed as protective antigens [27], are heavily glycosylated and may carry conformational epitopes that are not preserved when expressed in *E. coli*. To address this issue, we generated two replication-deficient VSV-based vaccine candidates, with the glycoprotein (G) replaced by either CD2v or EP153R (VSVΔG-CD2v and VSVΔG-EP153R; Figure 3A). We confirmed expression of the foreign proteins by intracellular staining of VSV-infected cells with serum from an ASFV-immune animal (Figure 3B and Figure S5B).

### 3.4. The Vaccines Did Not Induce Protection Against the Challenge Infection in Pigs

**Figure 4:**
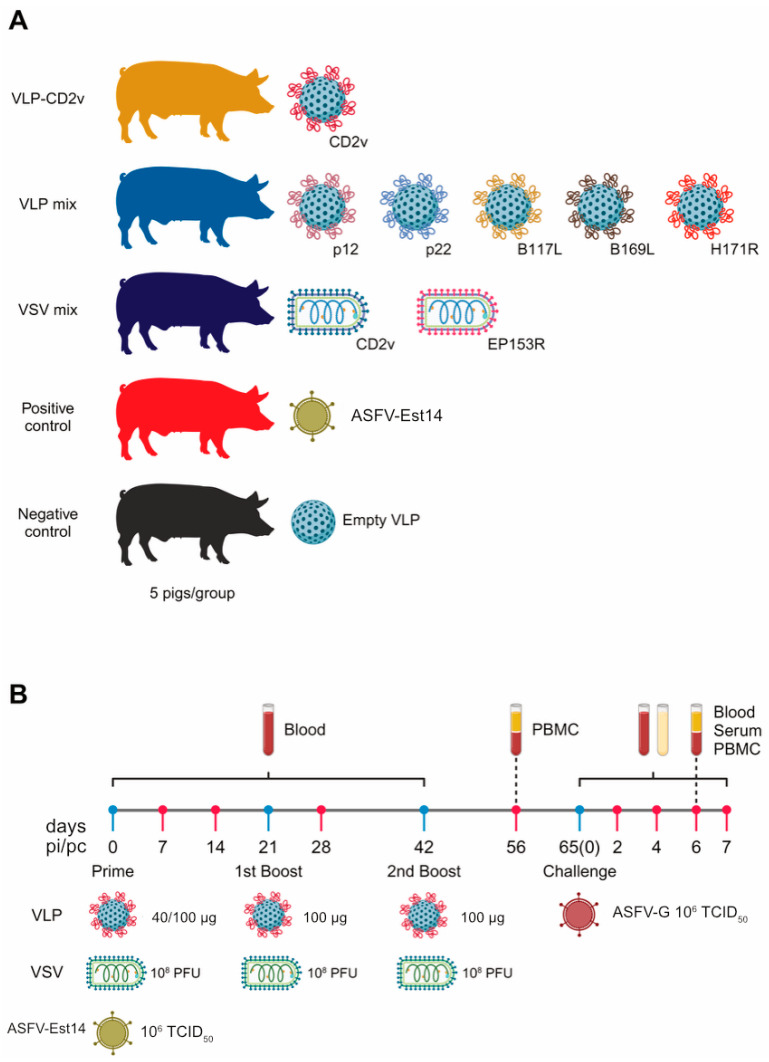
Experimental design of the vaccination-challenge experiment. (**A**) Vaccination and control groups. Five groups of animals (5 pigs/group) were included in the study. Three groups were immunized with either VLP- or VSV-based vaccines. The positive control group was infected with the attenuated ASFV-Est14 strain, while the negative control group received empty VLP. (**B**) Overview of the vaccination-challenge experiment. Pigs received three doses of either VLP- or VSV-based vaccines. ASFV-Est14 was administered once. After prime vaccination, blood samples were collected every three weeks in VLP and VSV groups. Blood samples were collected weekly from the positive control group for viral DNA measurement and hematological analysis. On day 65 (day 0 post-challenge), pigs were infected with the highly virulent ASFV-G strain. PBMC were collected before and after challenge to analyze cellular immune responses. Serum samples were used to measure cytokine and antibody responses, as well as to quantify viral DNA.

As a next step, we tested the ability of the generated vaccine candidates to confer protective immunity against the highly virulent ASFV-G strain in SPF pigs. The first group received VLPs displaying the soluble part of CD2v. The second group was injected with a mix of VLPs carrying either p12, p22, B117L, B169L, or H171R. The third group received a mix of VSVΔG-CD2v and VSVΔG-EP153R vectors. As a positive control for protection, one group of pigs was immunized with the attenuated ASFV-Est14 strain. The negative control group received empty VLPs composed of the scaffold protein (Figure 4A). The pigs were immunized three times with VLPs and VSVs, with a three-week interval between each dose. The challenge infection was performed on day 65 after the prime vaccination (Figure 4B).

**Figure 5:**
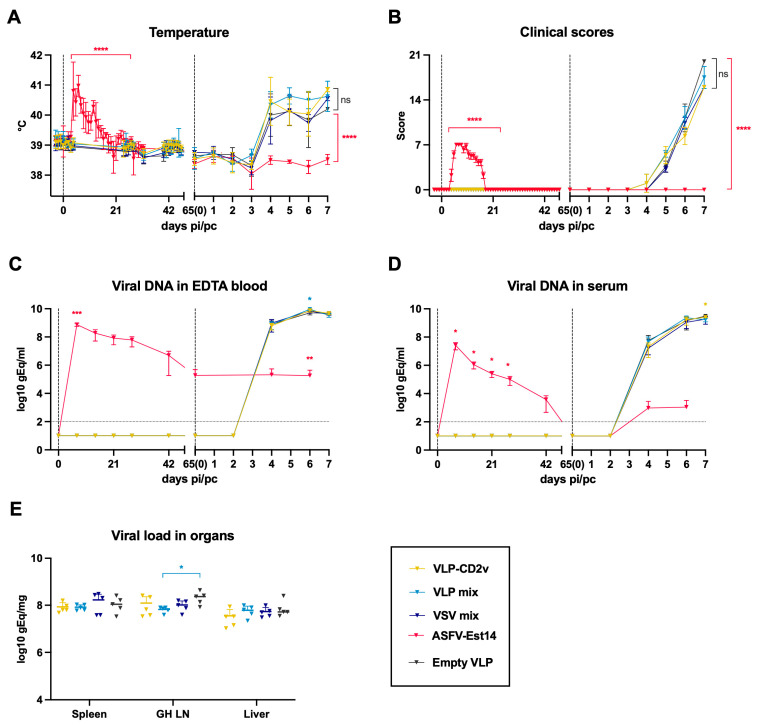
Outcomes of immunization and challenge. (**A**,**B**) Rectal temperatures and clinical scores. (**C**–**E**) ASFV DNA levels measured in genome equivalents (gE) in blood, serum and organs, respectively. Vertical dash lines mark time points of prime vaccination and challenge. Horizontal dotted lines show a cut-off value for negative qPCR results. For (**A**–**D**), the data points represent mean ± standard deviation of measurements. For (**E**), the data points represent values from individual animals and lines show mean ± standard deviation of measurements. For (**A**,**B**), area under curve (AUC) of both parameters for each vaccine group was compared to empty VLP group (immunization and challenge phases separately) by one-way ANOVA with Dunnett’s correction; ns—non-significant, **** *p* < 0.0001. For (**C**,**D**), values for each group were compared to empty VLP group at each time point using unpaired *t*-test with Holm–Šídák’s correction for multiple comparisons; * *p* < 0.05; ** *p* < 0.01; *** *p* < 0.001. For (**E**), the differences in viral load in each organ were analyzed by one-way ANOVA with Dunnett’s correction; * *p* < 0.05. No outliers in any of the datasets were identified.

The groups receiving VLP- or VSV-based vaccines did not exhibit any clinical symptoms such as fever and injection site reactions like swelling and discoloration during the immunization phase. Unfortunately, none of the animals in these vaccine groups were protected against the challenge, developing clinical symptoms with similar onset and severity as those in the negative control group that received empty VLPs. All pigs in the VLP and VSV groups reached the termination clinical score by 7 dpc (Figure 5A,B). In contrast, the animals in the ASFV-Est14 group exhibited moderate disease symptoms during immunization but fully recovered by 21 dpi; these pigs were fully protected against the challenge infection. Consistent with this, they developed viremia, which peaked at 7 dpi, followed by a gradual decline, eventually becoming undetectable in serum by 65 dpi. After the challenge, viral DNA levels remained unchanged in the blood of the positive control pigs and were slightly elevated in the serum. This contrasted with a dramatic increase in viral DNA levels in the VLP and VSV groups that were almost identical to the values of the negative control group (Figure 5C,D). We also did not observe significant differences in viral loads from the collected organs between the VLP- or VSV-vaccinated groups and the negative control. The only exception was a mild decrease in viral load in the gastrohepatic lymph nodes of animals in the VLP mix group (Figure 5E).

### 3.5. Strong Pro-Inflammatory Response in Non-Protected Animals upon Challenge

**Figure 6:**
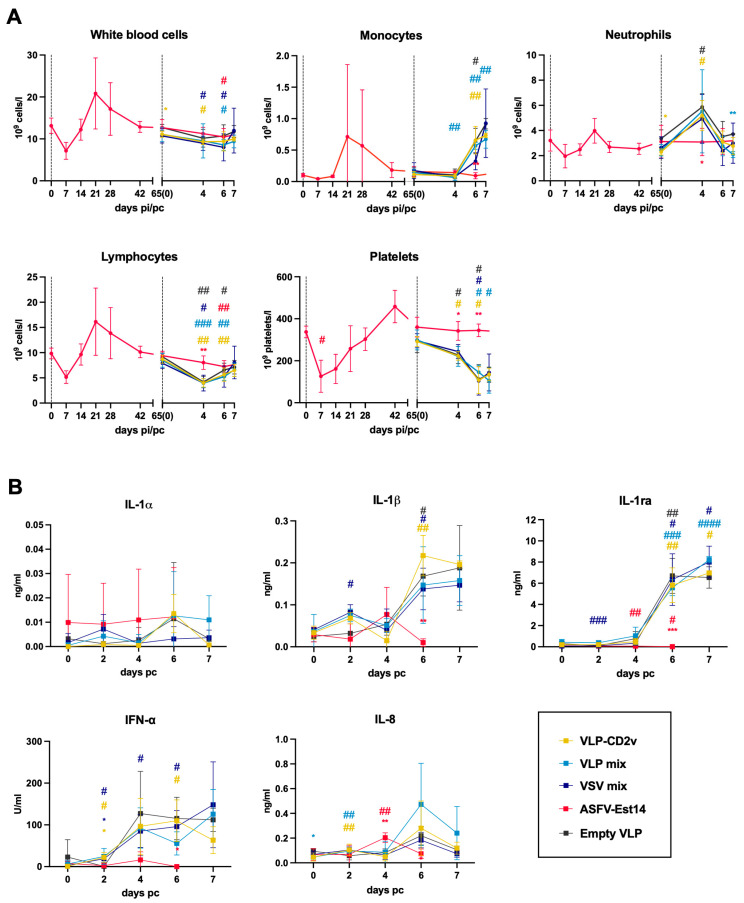
Hematological and cytokine profiles after immunization and challenge. (**A**) White blood cell, monocyte, neutrophil, lymphocyte, and platelet counts. Only samples from the ASFV-Est14 group were analyzed during the immunization phase. (**B**) Serum cytokine levels were measured by ELISA. Data points represent the mean ± standard deviation. Differences to baseline measurements (day 0 or day 65) for ASFV-Est14 group were analyzed by two-way ANOVA with Dunnett’s correction. Differences to baseline measurements (day 65) for VLP and VSV groups were analyzed by mixed-effects analysis with Dunnett’s correction; # *p* < 0.05; ## *p* < 0.01; ### *p* < 0.001; #### *p* < 0.0001. The statistically significant difference in IL-1RA at 2 dpc was of limited magnitude, driven by low inter-animal variability rather than a large biological effect. Differences for each group and individual cell subsets or cytokines were compared to empty VLP group at each time point using unpaired *t*-test with Holm–Šídák’s correction for multiple comparisons; * *p* < 0.05; ** *p* < 0.01; *** *p* < 0.001. No outliers in any of the datasets were identified.

We evaluated hematological and cytokine profiles of vaccinated pigs to gain insights into the immune responses that are positively or negatively associated with protection. In general, non-protected animals from the VLP and VSV groups exhibited uncontrolled pro-inflammatory responses like those of the negative control group (Figure 6A). We observed a significant decline in lymphocyte and platelet counts in non-protected animals starting from 4 dpc. In contrast, the numbers of monocytes and neutrophils increased, indicating an inflammatory response. The blood cell profiles of pigs in the ASFV-Est14 group exhibited a distinct pattern compared with the other groups. Although the animals experienced lymphopenia and recruitment of monocytes and neutrophils into the blood after immunization with the attenuated strain, leukocyte and platelet counts remained largely unchanged following the challenge.

The inflammatory response in non-protected animals after the challenge was further confirmed by analyzing serum cytokine levels (Figure 6B). Most notably, animals with high clinical scores at 6 and 7 dpc had significantly elevated levels of IL-1β and IL-1RA. The levels of IFN-α and IL-8 were also increased in most non-protected animals from 4 and 6 dpc, respectively, although with greater variability. Unlike the severely sick pigs, ASFV-Est14 group showed only a transient IL-8 response at 4 dpc and no pro-inflammatory cytokine response from 6 dpc onward.

### 3.6. Effective T Cell-Mediated Responses Are Associated with Protection

**Figure 7:**
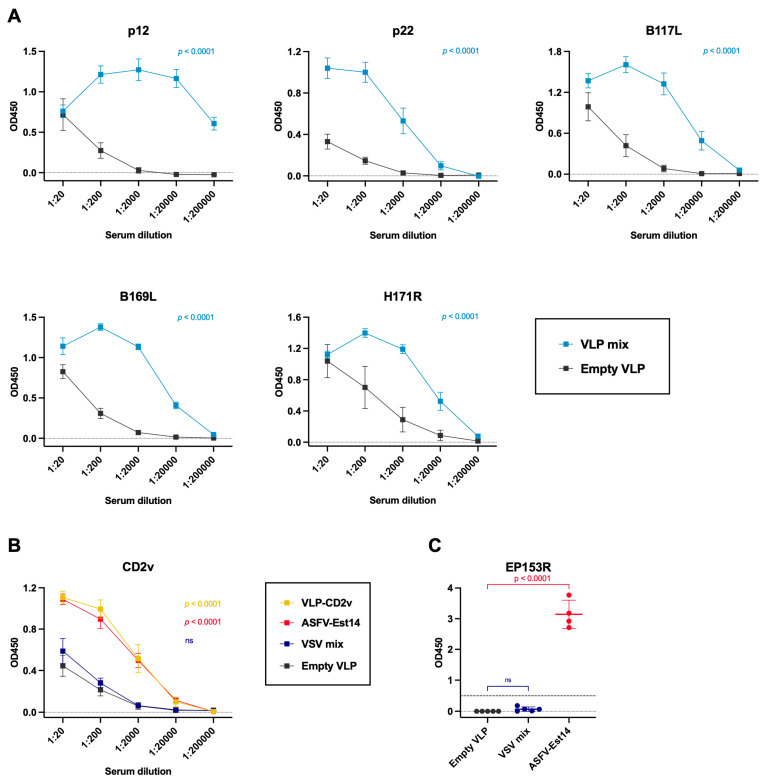
Serum antibody responses induced by the vaccine candidates measured by ELISA on day 65 post-immunization. OD values are shown (*y*-axis). (**A**) Antigen-specific antibody levels for the VLP mix. (**B**) CD2v-specific antibodies of the VLP-CD2v and VSV mix groups. (**C**) EP153R-specific antibodies in the VSV mix vaccines and ASFV-Est14 immunized pigs. For A and B, 10-fold serum dilutions were made (a-axis). For (**C**), sera were diluted 1:100. Data represent mean ± standard deviation of measurements. For statistical analyses of (**A**,**B**), the areas under the curve (AUC) of OD_450_ values were used and tested using unpaired *t* tests. For (**C**), vaccinated groups were compared to empty VLP group by one-way ANOVA with Dunnett’s correction for multiple comparisons; ns—non-significant. No outliers in any of the datasets were identified.

Next, we examined adaptive immune responses in vaccinated pigs. The antibody response to ASFV antigens used for VLP and VSV generation was evaluated prior to the challenge, on day 65 following the prime immunization. The animals in the VLP mix group demonstrated robust antibody production against six viral antigens (Figure 7A,B). Nevertheless, antibody levels against the CD2v and EP153R proteins in the VSV mix group were not significantly different from those of the empty VLP group. In contrast, animals in the ASFV-Est14 group developed strong humoral immune responses against these two antigens (Figure 7B,C).

**Figure 8:**
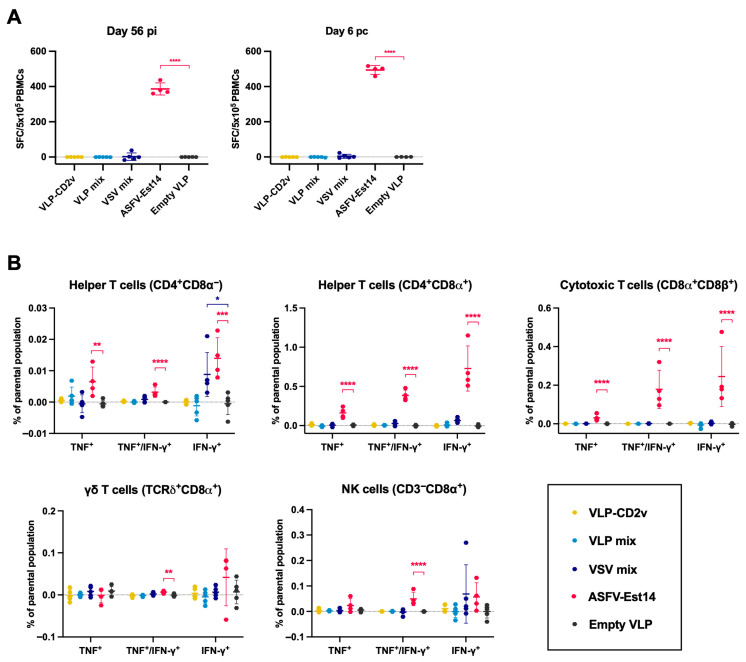
T-cell and NK cell responses following in vitro restimulation of PBMC with ASFV-Est14 on day 56 post-prime immunization. (**A**) IFN-γ ELISPOT-based detection of IFN-γ secreting cells as spot-forming cells (SFC) per 10^5^ PBMC (*y*-axis), which were isolated from the five different vaccine groups (*x*-axis). (**B**) IFN-γ and TNF responses detected by multicolor flow cytometry in five immune cell subsets (for gating strategy see Supplementary Figure S6). The *x*-axis shows the grouping of the cell subsets by cytokine production. The *y*-axis shows the frequency of cytokine-producing cells relative to the gated subsets. Data points represent values for individual animals, and lines indicate means ± standard deviations. Differences between vaccinated groups and negative control (empty VLP group) were analyzed by one-way ANOVA with Dunnett’s correction; * *p* < 0.05, ** *p* < 0.01, *** *p* < 0.001, **** *p* < 0.0001. No outliers in any of the datasets were identified.

Furthermore, we assessed IFN-γ secretion from freshly collected PBMCs upon virus restimulation before and after challenge (Figure 8A). Only the animals from the ASFV-Est14 group showed robust cytokine secretion. Additionally, the production of TNF and IFN-γ in five immune cell subsets following virus restimulation was analyzed by flow cytometry (Figure 8B and Figure S7). Substantial cytokine responses were detected before and after challenge only in the ASFV-Est14 group and were mediated by helper T cells (most prominent in the CD4^+^CD8α^+^ subset) and CD4^−^CD8α^+^CD8β^+^ cytotoxic T cells. Interestingly, a moderate IFN-γ production by CD4^+^CD8^−^ T cells was also observed in the VSV group, but the overall frequency in the Th cell populations compared to the ASFV-Est14 group remained very low. Effector γδ T cells remained largely inactive in all animal groups, while NK cells in the ASFV-Est14 group produced cytokines at 6 dpc.

## 4. Discussion

The development of safe and effective vaccines remains a primary goal in ASFV research. LAVs generated through targeted gene deletion have been shown to be effective against highly virulent genotype II strains. Nevertheless, their use is restricted due to safety concerns [2]. Alternative types of vaccines with improved safety profiles, such as viral vector-based, subunit, or DNA vaccines, have been developed and tested against both genotype I and II strains, but their efficacy is lower compared with that of LAVs, and needs targeting of multiple antigens [45]. Indeed, pools of adenovirus vector-based vaccine candidates encoding several ASFV genes have been demonstrated to confer protection against fatal disease following challenge with virulent genotype I ASFV [46,47]. This provides the foundation for ongoing efforts to develop safer vaccines against the currently circulating genotype II ASFV.

The present study focused on antibody-based protection by using vaccine delivery platforms known to promote strong B-cell responses. To identify potentially relevant antigens, we used immune sera from farm and SPF pigs, the latter of which have been shown to be more resilient to ASFV and develop a more robust immunity against challenge infection [23,48]. Accordingly, we hypothesized that this enhanced protection may be partially mediated by a broader spectrum of the antibody response. Indeed, the number of recognized proteins was higher when sera from SPF pigs were used for screening. Of the nine proteins detected by both farm and SPF sera, most are well-characterized antigens that have been evaluated in vaccines, with outcomes dependent on the specific formulation and delivery system employed [9]. On the other hand, many additional proteins identified by sera from SPF pigs or post-challenge infection have not been tested so far. For most of these proteins, our data confirm other antigen screening studies [49,50,51,52], except for DP79L and the unannotated ASFV-G_ACD_00320 and ACD_00600 proteins. Our data largely align with previous antigen screening studies [49,50,51,52], but we also identified several novel antigens not reported before, including I196L, H124R, B263R, MGF100-1L, B407L, DP79L and the unannotated ASFV-G_ACD_00320 and ACD_00600. 

Unfortunately, none of our candidate vaccines was able to reduce clinical disease, systemic pro-inflammatory, lymphocytopenia, and thrombocytopenia that were all induced by virulent ASFV genotype II challenge. There are several possible explanations for this negative result. The first could be that protection requires a high level of ASFV-specific memory T-cell generation, which was not induced by our vaccine candidates. T cells represent a correlate of protection against ASFV [53], and their importance is supported by many studies [3,54]. Thus, viral vaccine platforms that induce robust T-cell immunity, such as those based on adenovirus, should be preferred for ASFV [46]. Given that our approach focused on a possible contribution of antibodies, we opted for platforms that are less strong at inducing T-cell immunity, and T-cell epitopes were not considered for antigen selection. It should be noted that this induction of ASFV-specific T cells does not guarantee a protective effect, and a protective role for antibodies remains likely [47,55]. While for ASFV genotype I antibody transfer mediated at least partial protection [13], this has not been successfully demonstrated for genotype II [56]. Accordingly, an important knowledge gap is to investigate if and how antibodies contribute to protection against genotype II.

Assuming that antibodies would mediate partial protection against ASFV genotype II, which was our starting hypothesis, another possible explanation for the failure of our vaccine candidates was the selected antigens, as well as the type and height of humoral immunity induced, which was insufficient for protective immunity against genotype II ASFV. Considering that the ELISA data indicated a high level of antibody response against all selected proteins when using the VLP platform, we concluded that the induced antibodies were unable to mediate functional effects that would inhibit the virus.

Given that baculovirus-expressed CD2v can confer protection against virulent genotype I ASFV [57], our results indicate that a subunit vaccine based on VLP-CD2v alone is insufficient for protection. This may be due to epitope loss following expression as CD2v-Fc fusion protein on the VLP and/or to the use of solely the soluble extracellular domain of CD2v for VLP generation, as the intracellular domain also contains B- and T-cell epitopes [58].

In view of the robust antibody responses against p12, p22, B117L, B169L, and H171R in the VLP mix group, we conclude that these five antigens are not protective when delivered on VLP. To our knowledge, there is no report on using these proteins in a vaccine approach. Both p22 and p12 have been found on the cell surface or associated with infected cells [42,59], with p12 being involved in virus attachment [60]. Further investigations are required to test possible genotype differences in this localization. Furthermore, monospecific antibodies should be tested for possible FcR-dependent antibody functions such as ADCC. As such sera were not available and our data indicated a complete lack of protection, we did not perform such assays. The antigens B117, B169L and H171R were more exploratory and based on our data, we propose that they should not be included in vaccine candidates.

For CD2v and EP153R, which are both heavily glycosylated and expressed on the surface of infected cells [17], we utilized replication-deficient VSV vectors as an alternative strategy for antigen delivery to ensure presentation of epitopes in their native conformation. We considered these two proteins as promising candidates as they define ASFV serotypes and are expressed on the surface of infected cells, and for CD2v also in the outer envelope [17,26,27,28,61]. Therefore, antibodies against these proteins could potentially exhibit neutralizing activity, ADCC, ADCP, and even complement-dependent cytotoxicity. In the present study, we confirmed the expression of two proteins in mammalian cell lines, supporting the assumption that they would be presented to the immune system in their native forms when expressed by VSV vectors in pigs. A possible explanation for the lack of protection is that the vaccines induced very low antibody responses and no detectable T-cell activation compared with those observed in the positive control group. As the VSV vector was previously found to induce high antibody levels in pigs [21], we think that the heavy glycosylation might act as a glycan shield, reducing their immunogenicity. Based on this experience, it might be recommended to use replication-competent viral vectors that ensure a longer antigen persistence to ensure several rounds of affinity maturation by somatic hypermutation mimicking LAV [62].

Taken together, the present work provides a comprehensive list of immunogenic antigens, identified after primary immunization and expanded after challenge infection, which will be helpful for future diagnostics and vaccine design. The selection of vaccine antigens based on their recognition only with sera from protected pigs was not successful and might simply be an effect of the higher T helper cell activity driving a broader B cell response in SPF pigs [53]. Based on our experience, we propose that future screening for antigens should include analyses of antibody functions such as assays for neutralization, ADCC, ADCP, antibody-dependent enhancement of infection, complement-mediated antibody functions and hemagglutination inhibition. Furthermore, while the lack of protection is informative for future work, our data suggest that effective ASF vaccines will require the integration of appropriately selected antigens with delivery platforms capable of inducing both functionally relevant antibodies and potent T-cell responses.

## Data Availability

The data generated in the present study may be requested from the corresponding author.

## Supplementary Materials

The following supporting information can be downloaded at: https://www.mdpi.com/article/10.3390/vaccines14030285/s1, Supplementary Figure S1: Microarray construction; Supplementary Figure S2: Amino acid sequences of VLP and VSV constructs; Supplementary Table S1: List of antibodies used for flow cytometry; Supplementary Figure S3: SDS-PAGE analysis of AP205d-fusion proteins; Supplementary Figure S4: SDS-PAGE images of AP205d-fusion proteins; Supplementary Figure S5: ASFV protein expression in VSV-infected HEK-293 and IPKM cells; Supplementary Figure S6: Gating strategy for analysis of intracellular cytokine responses following in vitro restimulation of freshly collected PBMCs with the live virus; Supplementary Figure S7: Cellular immune responses following in vitro restimulation of PBMCs with ASFV on day 6 post-challenge.
