# Supplementary material for: Virus-like and Virus Replicon Particles Targeting Multiple B-Cell Antigens Do Not Protect Against African Swine Fever Virus
Source: Vaccines (Basel). 2026 Mar 23;14(3):285. doi: 10.3390/vaccines14030285 (PMC13030806; doi:10.3390/vaccines14030285)
Supplement: Supplementary file 1 [file vaccines-14-00285-s001.zip › vaccines-4175063-supplementary.pdf]

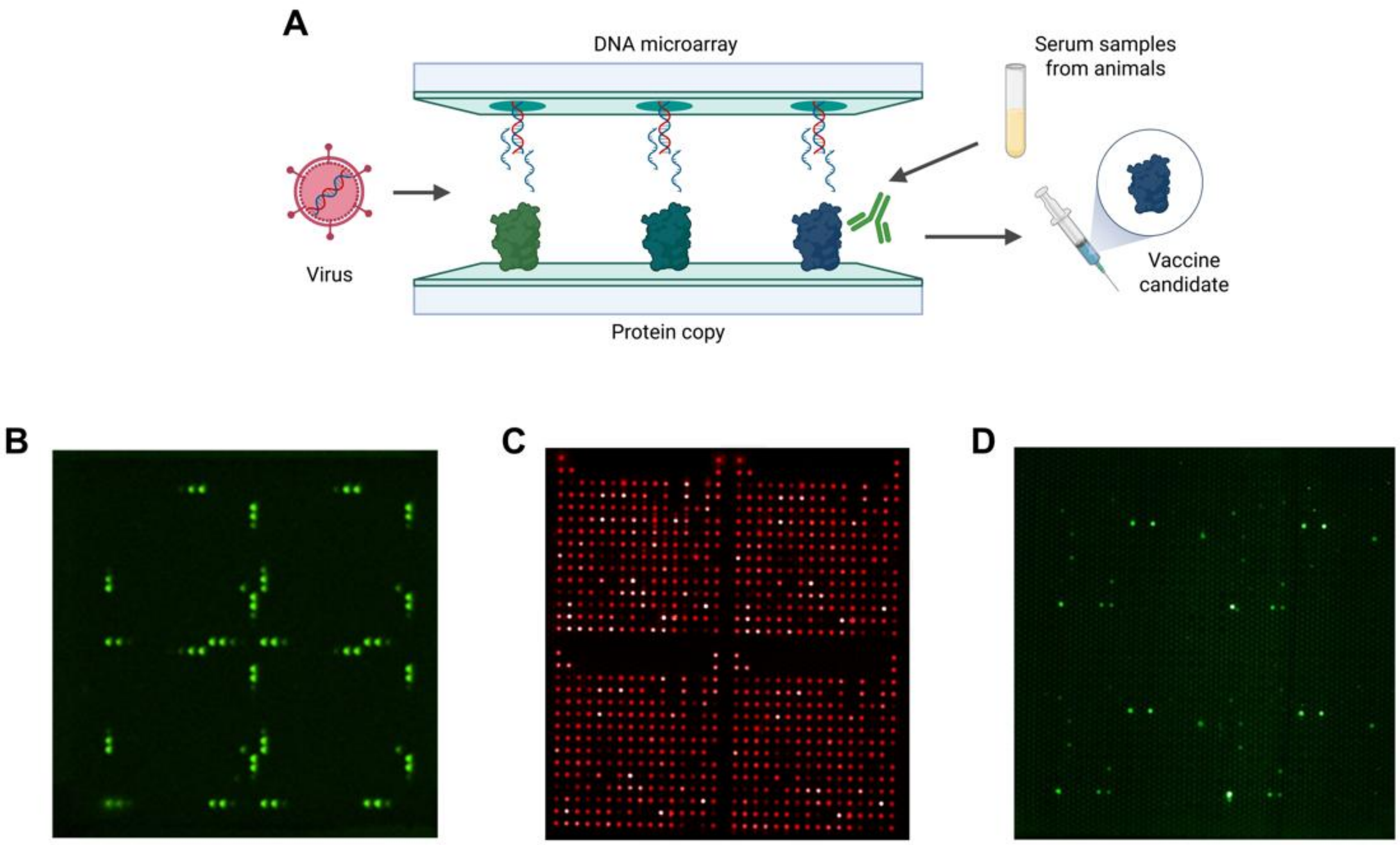


**Supplementary Figure 1.** Microarray construction. (**A**) Pipeline of antigen identification established by BioCopy (adapted from www.biocopy.com). (**B**) Verification and quality control of protein expression via detection of GFP fluorescence from control spots. (**C**) Microarray showing expression of HA-tagged ASFV proteins (red signal), with each quadrant representing a replicate of all expressed proteins. (**D**) Representative data for detection of antigenic ASFV proteins (green signal) on the microarray chip.

p12 (peptide) MALDGSSGGGSN

p22    GHHHHHHENLYFQSKKQQPPKKVCKVDKDCGSGEHCVRGSCSSLSCLDAVKMDKRNIKIDSKISSCEFTPNFYRFTD
TAADEQQEFGKTRHPIKITPSPSESHSPQEVCEKYCSWGTDDCTGWEYVGDEKEGTCYVYNNPHHPVLKYGKDHIIALPRN
HKHA

AP205-B169L  MANKPMQPITSTANKIVWSDPTRLSTTFSASLLRQRVKVGIAELNNVSGQYVSVYKRPAPKPEGCADACVIM
PNENQSIRTVISGSAENLATLKAEWETHKRNVDTLFASGNAGLGFLDPTAAIVSSDTTAGSANKPMQPITSTANKIVWSDPTR
LSTTFSASLLRQRVKVGIAELNNVSGQYVSVYKRPAPKPEGCADACVIMPNENQSIRTVISGSAENLATLKAEWETHKRNVD
TLFASGNAGLGFLDPTAAIVSSDTTANHQLNDIYNKSNMDVIVSSIHDKYKGGDEIIPPISPPSVSNELEEDQPKKIPAGPKPAD
SKPVSLPDSKPLVPLQEVIMPSQYNN

AP205-B117L  MANKPMQPITSTANKIVWSDPTRLSTTFSASLLRQRVKVGIAELNNVSGQYVSVYKRPAPKPEGCADACVIM
PNENQSIRTVISGSAENLATLKAEWETHKRNVDTLFASGNAGLGFLDPTAAIVSSDTTAGSANKPMQPITSTANKIVWSDPTR
LSTTFSASLLRQRVKVGIAELNNVSGQYVSVYKRPAPKPEGCADACVIMPNENQSIRTVISGSAENLATLKAEWETHKRNVD
TLFASGNAGLGFLDPTAAIVSSDTTAGYTIQLDKDGDYCWDEDPTHHDPYMQANATSHVATSYATTSHAATPHAAAHHTFHE
PFIKLNLTDKN

AP205-H171R MANKPMQPITSTANKIVWSDPTRLSTTFSASLLRQRVKVGIAELNNVSGQYVSVYKRPAPKPEGCADACVIM
PNENQSIRTVISGSAENLATLKAEWETHKRNVDTLFASGNAGLGFLDPTAAIVSSDTTAGSANKPMQPITSTANKIVWSDPTR
LSTTFSASLLRQRVKVGIAELNNVSGQYVSVYKRPAPKPEGCADACVIMPNENQSIRTVISGSAENLATLKAEWETHKRNVD
TLFASGNAGLGFLDPTAAIVSSDTTAGSGVVYDLLVSLSKESIDVLRFVEANLAAFNQQYIFFNIQRKNSITTPLLITPQQEKISQ
IVEFLMDEYNKNNRRPSGPPREQPMHPLLPYQQSSDEQPMMPYQQPPGNDDQPYEQIYHKKHASQQVNTELNDYYQHIL
ALGDEDKGMDSMLKLPEKAKRDSDDEDDMFSIKN

AP205d-CD2v-Fc  ATMGILPSPGMPALLSLVSLLSVLLMGCVAGHHHGSGGSGCDYWVSFNKTIILDSNITNDNNDINGVSWN
FFNNSFNTLATCGKAGNFCECSNYSTSIYNITNNCSLTIFPHNDVFDTTYQVVWNQIINYTIKLLTPATPPNITYNCTNFLITCKK
NNGTNTNIYLNINDTFVKYTNESILEYNWNNSNINNFTATCIINNTISTSNETTLINCTYLTLSSNYFYTFFKLYGGCGGENLYFQ
SDKTHTCPPCPAPELLGGPSVFLFPPKPKDTLMISRTPEVTCVVVDVSHEDPEVKFNWYVDGVEVHNAKTKPREEQYNSTY
RVVSVLTVLHQDWLNGKEYKCKVSNKALPAPIEKTISKAKGQPREPQVYTLPPSRDELTKNQVSLTCLVKGFYPSDIAVEWE
SNGQPENNYKTTPPVLDSDGSFFLYSKLTVDKSRWQQGNVFSCSVMHEALH NHYTQKSLSLSPGK

pVSV-dG-EP402R ATGATAATACTTATTTTTTTAATATTTTCTAACATAGTTTTAAGTATTGATTATTGGGTTAGTTTTAATAAAA
CAATAATTTTAGATAGTAATATTACTAATGATAATAATGATATAAATGGAGTATCATGGAATTTTTTTAATAATTCTTTTAATACA
CTAGCTACATGTGGAAAAGCAGGTAACTTTTGTGAATGTTCTAATTATAGTACATCAATATATAATATAACAAATAATTGTAG
CTTAACTATTTTTCCTCATAATGATGTATTTGATACAACATATCAAGTAGTATGGAATCAAATAATTAATTATACAATAAAATTA
TTAACACCTGCTACTCCCCCAAATATCACATATAATTGTACTAATTTTTTAATAACATGTAAAAAAAATAATGGAACAAACAC
TAATATATATTTAAATATAAATGATACTTTTGTTAAATATACTAATGAAAGTATACTTGAATATAACTGGAATAATAGTAACATTA
ACAATTTTACAGCTACATGTATAATTAATAATACAATTAGTACATCTAATGAAACAACACTTATAAATTGTACTTATTTAACATT
GTCATCTAACTATTTTTATACTTTTTTTAAATTATATTATATTCCATTAAGCATCATAATTGGGATAACAATAAGTATTCTTCTTA
TATCCATCATAACTTTTTTATCTTTACGAAAAAGAAAAAAACATGTTGAAGAAATAGAAAGTCCACCACCTGAATCTAATGA
AGAAGAACAATGTCAGCATGATGACACCACTTCCATACATGAACCATCTCCCAGAGAACCATTACTTCCTAAGCCTTACA
GTCGTTATCAGTATAATACACCTATTTACTACATGCGTCCCTCAACACAACCACTCAACCCATTTCCCTTACCTAAACCGT
GTCCTCCACCCAAACCATGTCCGCCACCCAAACCATGTCCTCCACCTAAACCATGTCCTTCAGCTGAATCCTATTCTCC
ACCCAAACCACTACCTAGTATCCCGCTACTACCCAATATCCCGCCATTATCTACCCAAAATATTTCGCTTATTCACGTAGAT
AGAATTATTTAA

pVSV-dG-EP153R    ATGTTTTCTAACAAAAAGTACATCGGTCTTATCAATAAGAAGGAGGGTTTGAAAAAAAAAAATAGATG
ATTATAGTATATTAATAATTGGAATATTAATTGGAACTAACATCTTAAGCCTTATTATAAATATAATAGGAGAGATTAATAAACC
AATATGTTACCAAAATGATGATAAGATATTTTATTGCCCTAAAGATTGGGTTGGATATAATAATGTTTGTTATTATTTTGGCAA
TGAAGAAAAAAATTATAATAATGCAAGTAATTATTGTAAGCAATTAAATAGTACGCTTACTAATAATAATACTATTTTAGTAAAT
CTTACTAAAACATTAAATCTTACTAAAACATATAATCACGAATCTAATTATTGGGTTAATTATTCTTTAATTAAAAATGAGTCAG
TACTATTACGTGATAGTGGATATTACAAAAAACAAAAACATGTAAGTTTATTATATATTTGTAGTAAATAA

**Supplementary Figure 2.** Amino acid sequences of VLP and VSV constructs. Light blue – ASFV peptide or protein domain, brown – His-tag, green – TEV protease linker, violet – AP205, yellow – linker, pink – hFc.

| Antigen | Clone | Species/Isotype | Label | Source | Cat. No. |
|---|---|---|---|---|---|
| IgG-Fc* | Polyclonal | Goat IgG | Biotin | Bethyl Laboratories | A100-104B |
| CD3 | PPT3 | Mouse IgG1 | - | Hybridomas | - |
| CD4 | 74-12-4 | Mouse IgG2b | - | Hybridomas | - |
| CD8α | 76-2-11 | Mouse IgG2a | PE | BD Pharmingen | 559584 |
| CD8β | PG164A | Mouse IgG2a | - | WSU | PG164A |
| δ-TCR* | PGBL22A | Mouse IgG1 | - | WSU | PGBL22A |
| IFN-γ | P2G10 | Mouse IgG1 | PerCP-Cy5.5 | BD Pharmingen | 561481 |
| TNF-α | MAb11 | Mouse IgG1 | AF647 | BioLegend | 502916 |
| IgG1 | Polyclonal | Goat IgG | APC/CY7 | SouthernBiotech | 1070-19 |
| IgG2b | Polyclonal | Goat IgG | AF488 | Invitrogen | A-21141 |
| IgG2a | Polyclonal | Goat IgG | PE/Cy7 | Abcam | ab130787 |

**Supplementary Table 1.** List of antibodies used for flow cytometry. *The antibody against δ-TCR chain was coupled to biotin using Zenon Mouse IgG1 labeling kit (Z25052, Invitrogen, USA). Streptavidin coupled with BV421 was used as a conjugate (563259, BD Horizon, USA) to detect biotin-labeled antibodies.

**A**

| VLP-fusion protein | Relative band intensity |
|---|---|
| p12 (1) | 61.6% |
| p22 alone (2) | 43.1% |
| AP205 VLP (3) | 48.9% |
| AP205d-p22 (4) | 11.6% |
| H171R (5) | 49.1% |
| B169L (6) | 63.9% |
| B117L (7) | 35.0% |
| CD2v-Fc | 71.2% |
| CD2v-Fc (10 μL TEV) | 3.9% |
| CD2v-Fc (5 μL TEV) | 2.1% |

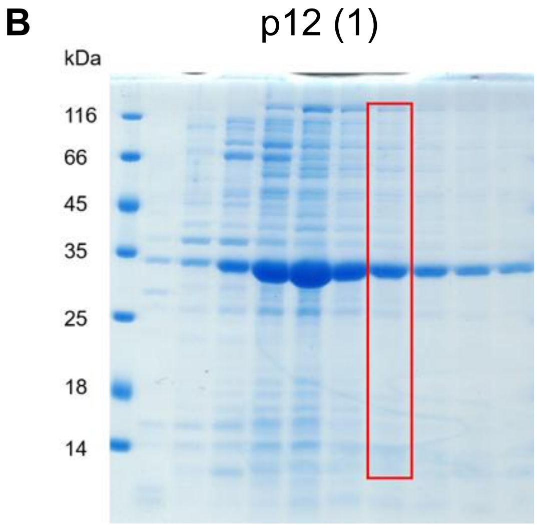


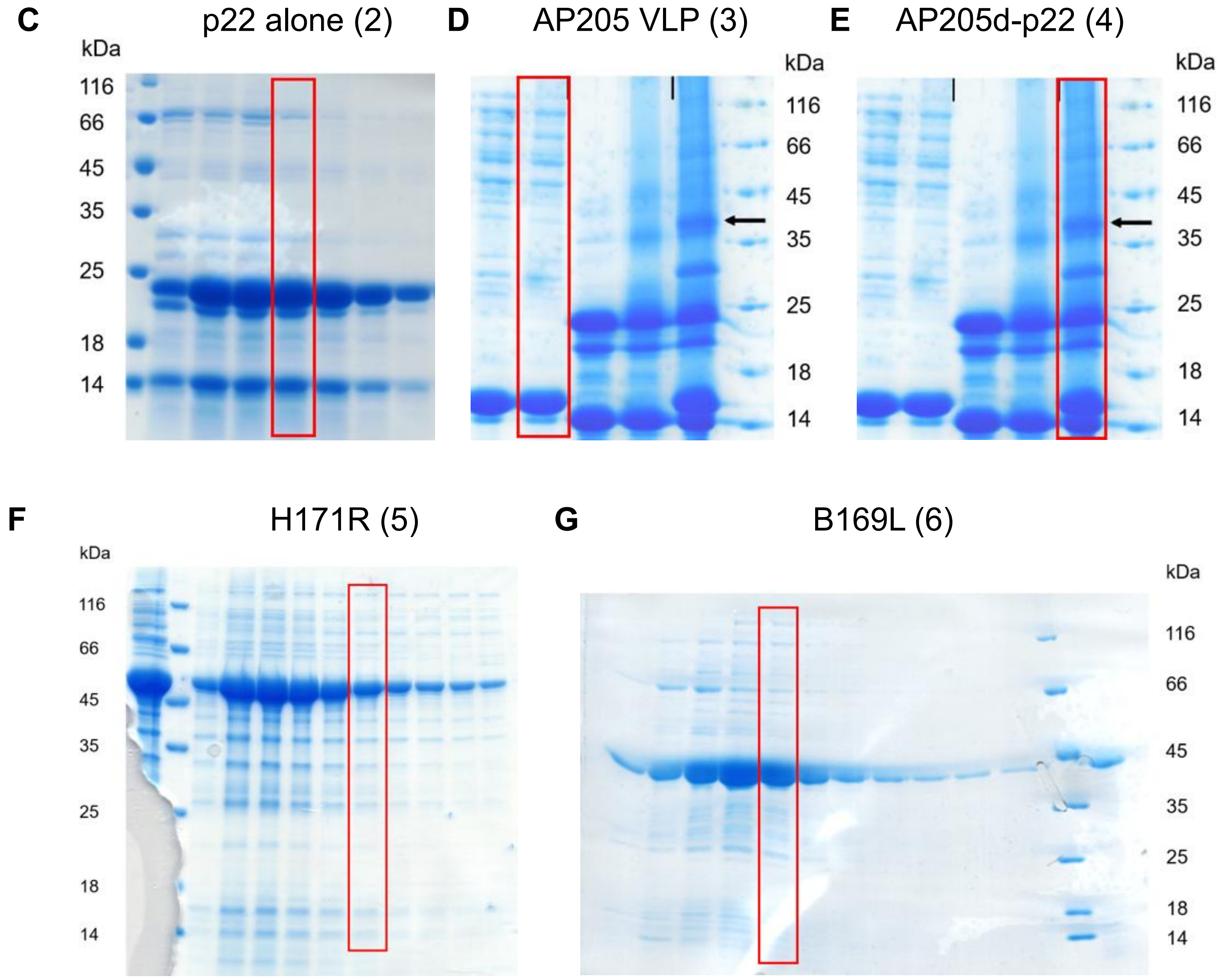


**Supplementary Figure 3.** SDS-PAGE analysis of AP205d-fusion proteins. (A) Relative band intensities were quantified by ImageJ densitometry and expressed as a fraction of the total lane signal. For protease-treated samples, the intensity of the cleaved band was normalized to total lane intensity. (B-G) The images are shown together with molecular weight markers. Lanes outlined in red correspond to those shown in Figure 2A. Numbers in brackets correspond to the lane labels in Figure 2A.

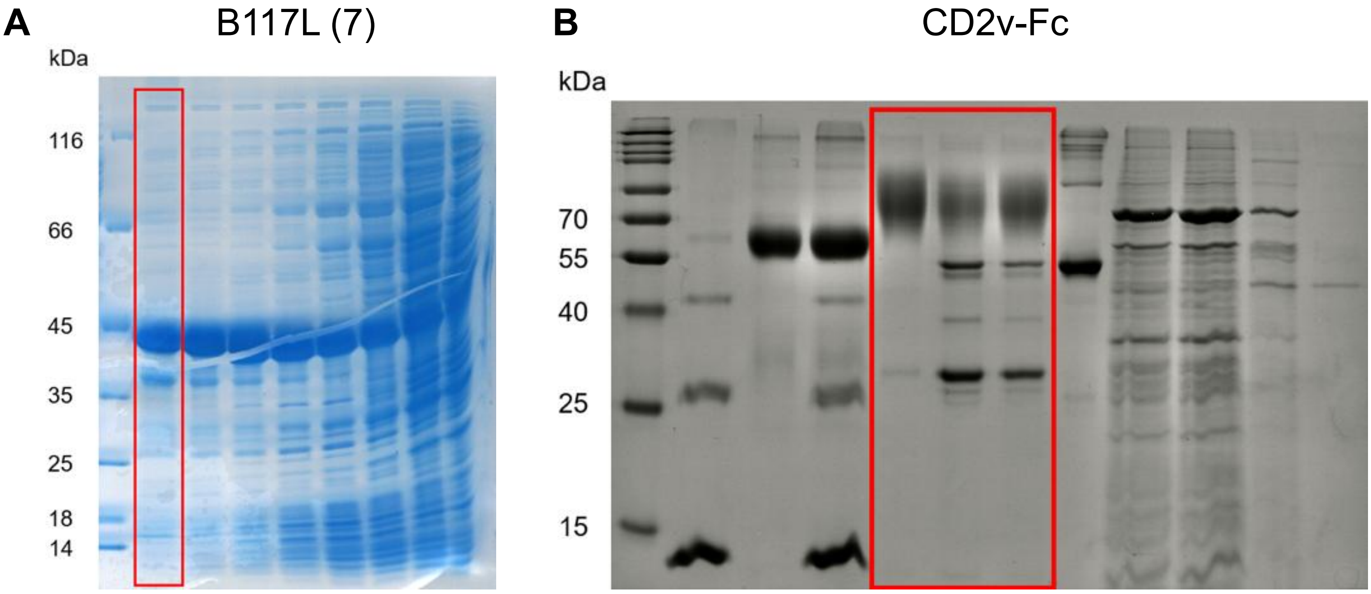


**Supplementary Figure 4.** SDS-PAGE images of AP205d-fusion proteins. (A, B) The images are shown together with molecular weight markers. Lanes outlined in red correspond to those shown in Figures 2A and 2B. Number in brackets corresponds to the lane label in Figure 2A.

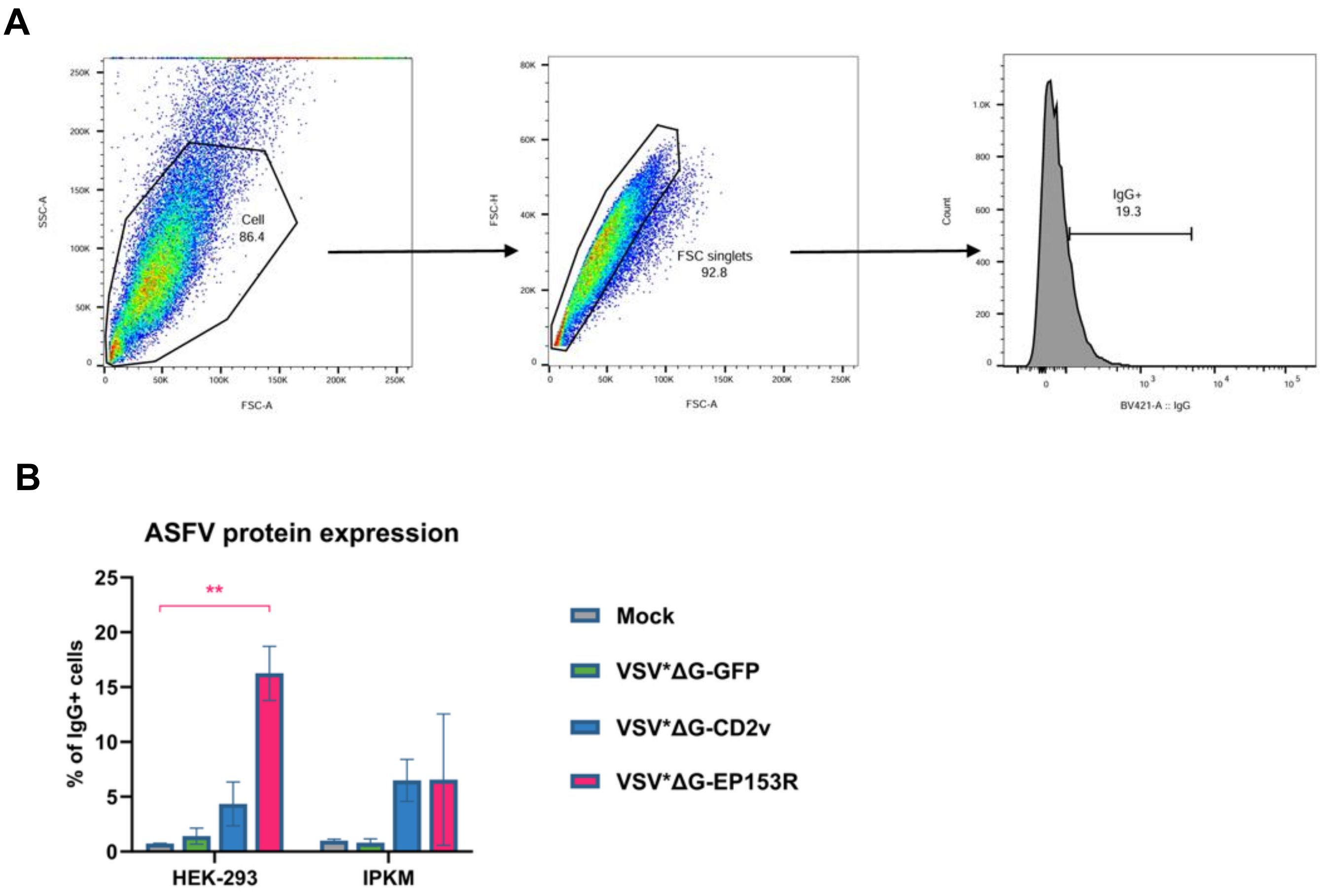


**Supplementary Figure 5.** ASFV protein expression in VSV-infected HEK-293 and IPKM cells. (A) Gating strategy for detection of CD2v and EP153R proteins. (B) The expression of ASFV proteins in VSV-infected cells was confirmed by staining with ASFV-positive serum. Data represent the mean ± standard deviation of measurements with duplicates. Statistical comparison was performed by one-way ANOVA with Dunnett's correction for multiple comparisons; **$p<0.01$.

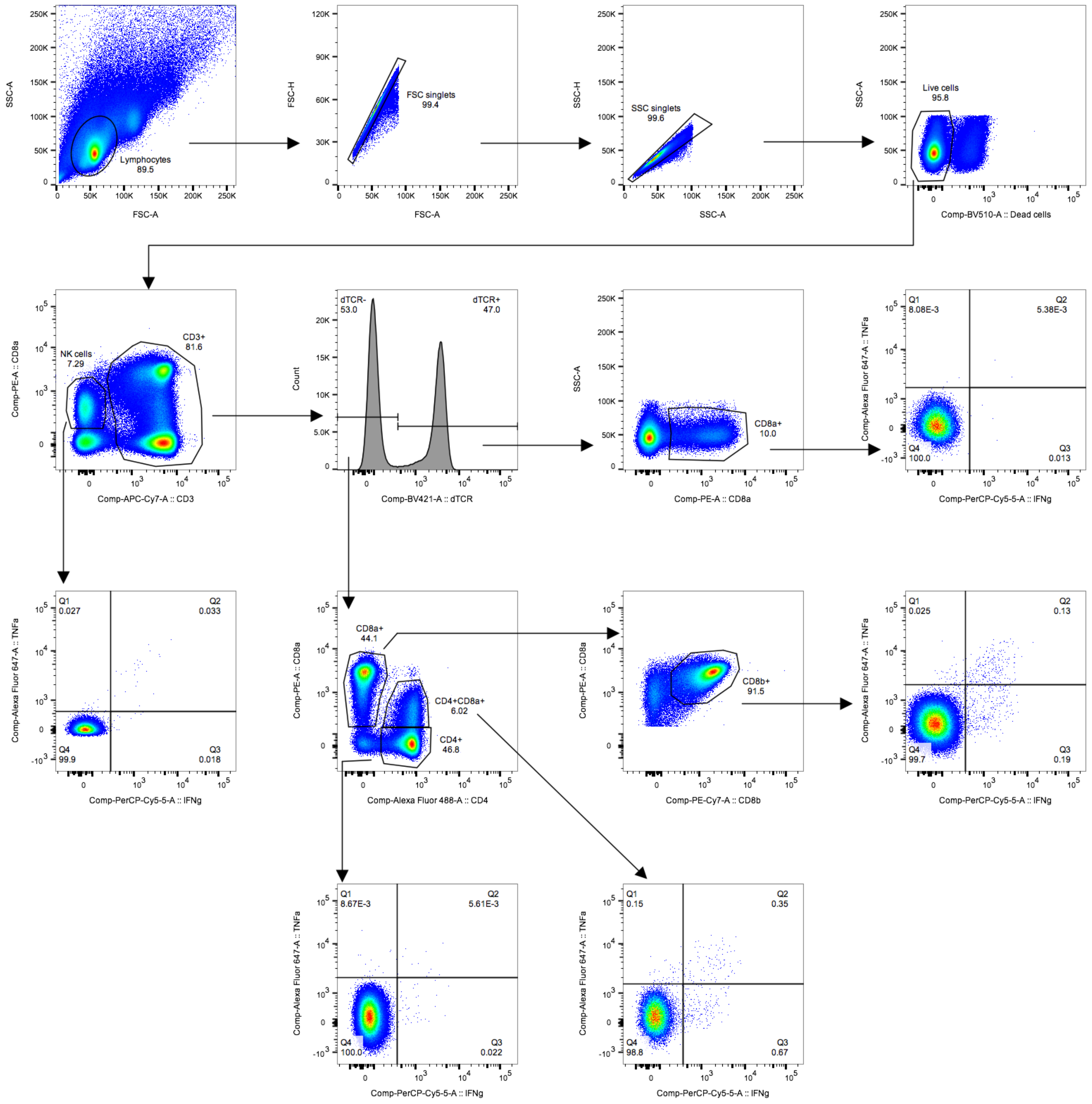


**Supplementary Figure 6.** Gating strategy for analysis of intracellular cytokine responses following in vitro restimulation of freshly collected PBMCs with the live virus.

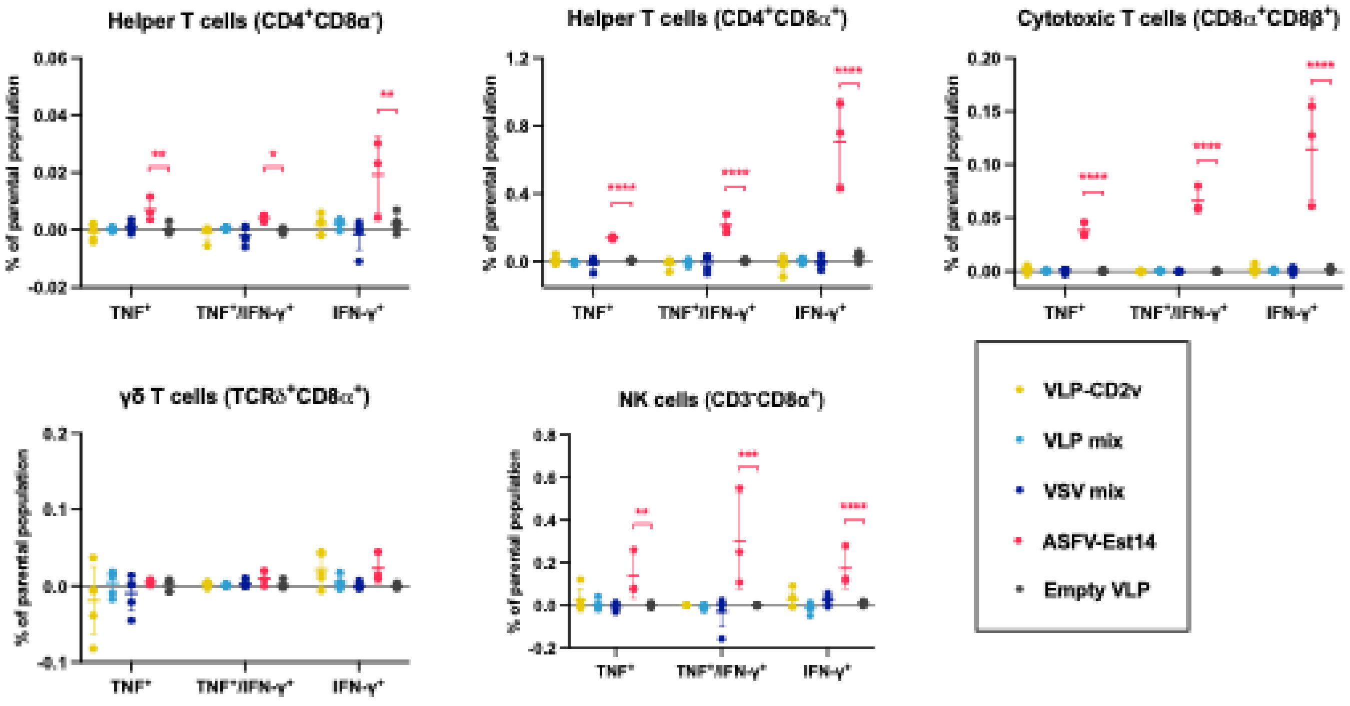


**Supplementary Figure 7.** Cellular immune responses following in vitro restimulation of PBMCs with ASFV on day 6 post-challenge. Data points represent values for individual animals. Lines indicate means ± standard deviations. Differences between vaccinated groups and negative control (empty VLP group) were analyzed by one-way ANOVA with Dunnett's correction; *p<0.05, **p<0.01, ***p<0.001, ****p<0.0001.
